# TBX2, a Novel Regulator of Labour

**DOI:** 10.3390/medicina57060515

**Published:** 2021-05-21

**Authors:** Febilla Fernando, Geertruda J.M. Veenboer, Martijn A. Oudijk, Marlies A.M. Kampman, Karst Y. Heida, Louise J.M. Lagendijk, Joris A.M. van der Post, Aldo Jongejan, Gijs B. Afink, Carrie Ris-Stalpers

**Affiliations:** 1 Reproductive Biology Laboratory, Amsterdam Reproduction and Development, Amsterdam UMC, University of Amsterdam, Meibergdreef 9, 1105 AZ Amsterdam, The Netherlands; febilla.fernando@gmail.com (F.F.); klapros117@hotmail.com (G.J.M.V.); louisejml@hotmail.com (L.J.M.L.); g.b.afink@amsterdamumc.nl (G.B.A.); 2 Department of Obstetrics and Gynaecology, Amsterdam Reproduction and Development, Amsterdam UMC, University of Amsterdam, Meibergdreef 9, 1105 AZ Amsterdam, The Netherlands; m.a.oudijk@amsterdamumc.nl (M.A.O.); j.a.vanderpost@amsterdamumc.nl (J.A.M.v.d.P.); 3 Department of Cardiology, University Medical Center Groningen, University of Groningen, Hanzeplein 1, 9713 GZ Groningen, The Netherlands; marlieskampman@hotmail.com; 4 Department of Obstetrics, Division of Woman and Baby, University Medical Center Utrecht, Heidelberglaan 100, 3584 CX Utrecht, The Netherlands; k.y.heida@gmail.com; 5 Department of Clinical Epidemiology, Biostatistics and Bioinformatics, Amsterdam UMC, University of Amsterdam, Meibergdreef 9, 1105 AZ Amsterdam, The Netherlands; a.jongejan@amsterdamumc.nl

**Keywords:** delivery, TERT-HM cells, inflammation, myometrium, premature delivery, progesterone withdrawal, proinflammatory cytokines and chemokines, TBX2, uterine smooth muscle cells

## Abstract

*Background and Objectives*: Therapeutic interventions targeting molecular factors involved in the transition from uterine quiescence to overt labour are not substantially reducing the rate of spontaneous preterm labour. The identification of novel rational therapeutic targets are essential to prevent the most common cause of neonatal mortality. Based on our previous work showing that Tbx2 (T-Box transcription factor 2) is a putative upstream regulator preceding progesterone withdrawal in mouse myometrium, we now investigate the role of TBX2 in human myometrium. *Materials and Methods*: RNA microarray analysis of (A) preterm human myometrium samples and (B) myometrial cells overexpressing TBX2 in vitro, combined with subsequent analysis of the two publicly available datasets of (C) Chan et al. and (D) Sharp et al. The effect of TBX2 overexpression on cytokines/chemokines secreted to the myometrium cell culture medium were determined by Luminex assay. *Results*: Analysis shows that overexpression of TBX2 in myometrial cells results in downregulation of TNFα- and interferon signalling. This downregulation is consistent with the decreased expression of cytokines and chemokines of which a subset has been previously associated with the inflammatory pathways relevant for human labour. In contrast, CXCL5 (C-X-C motif chemokine ligand 5), CCL21 and IL-6 (Interleukin 6), previously reported in relation to parturition, do not seem to be under TBX2 control. The combined bioinformatical analysis of the four mRNA datasets identifies a subset of upstream regulators common to both preterm and term labour under control of TBX2. Surprisingly, TBX2 mRNA levels are increased in preterm contractile myometrium. *Conclusions*: We identified a subset of upstream regulators common to both preterm and term labour that are activated in labour and repressed by TBX2. The increased TBX2 mRNA expression in myometrium collected during a preterm caesarean section while in spontaneous preterm labour compared to tissue harvested during iatrogenic preterm delivery does not fit the bioinformatical model. We can only explain this by speculating that the in vivo activity of TBX2 in human myometrium depends not only on the TBX2 expression levels but also on levels of the accessory proteins necessary for TBX2 activity.

## 1. Introduction

Preterm birth, defined as delivery before the 37th week of gestation, is the most common cause of neonatal mortality and the second leading cause of death in children under five years of age. Preterm birth is associated with immediate and long-term morbidity as well as growth and developmental delay [1,2,3]. The last 2 decades show increasing rates of preterm birth in almost all countries [4]. Although preterm birth can be iatrogenic, the majority of cases are due to spontaneous preterm labour. Currently there is no treatment that can prevent or block preterm labour. Known clinical risk factors for preterm labour include multiple pregnancy, polyhydramnios, cervical insufficiency, recurrent blood loss, previous preterm birth, infection/inflammation and placental ischaemia [1,5].

The characterised molecular mechanisms underlying the transition from uterine quiescence to synchronized contractions that allow delivery of the foetus are functional progesterone withdrawal and the stimulation of inflammatory pathways. The inhibition of progesterone receptor (PR) transcriptional activity in humans is governed by the PR-A (progesterone receptor-A-mediated inhibition of PR-B (progesterone receptor-B) transcriptional activity combined with decreased expression of ZEB1 (zinc finger E-box binding homeobox 1), ZEB2 (zinc finger E-box binding homeobox 1) and PR co-activator levels, increased levels of miR-200s and enhanced progesterone metabolism [6,7]. An in depth review by Renthal et al. describes the molecular mechanisms mediated by progesterone and oestradiol [8]. Functional progesterone withdrawal and the concomitant downstream effects are related to stimulation of the NFkB (nuclear factor kappa B)transcription factor complex that is activated in response to pro-inflammatory stimuli [9]. The combined action of inflammatory factors and functional progesterone withdrawal induces prostaglandin synthesis and activates the oestrogen receptor alpha and the oxytocin receptor that result in both increased oestrogen and oxytocin bioactivity [10,11]. The voltage gated Ca^2+^ channels that are subsequently activated facilitate uterine contractions while the increased expression of the gap-junction alpha-1 protein connexin 43 is essential to achieve synchronization of uterine myocyte activity [12]. Activation of the inflammatory pathways and progesterone withdrawal are clearly prerequisite for functional labour, but their interaction is complex [13]. It has been hypothesized that multiple intrinsic and extrinsic stressors whose effects are integrated and result in an inflammatory load on the uterine tissues may induce the functional progesterone withdrawal trigger for parturition [9,14,15]. Both the increased phosphorylation of PR-A at serine 345 associated with the onset of labour and the increased stability of PR-A are induced by interleukin-1β [16,17]. This currently available evidence suggests that inflammation precedes functional myometrial progesterone withdrawal in humans. Apart from the myometrium, also the placenta, the membranes and the cervix play an important role in the transition from uterine quiescence to labour [18,19].

Systematic reviews of clinical trials seem to indicate a modest reduction of preterm birth after progesterone treatment of women at-risk for preterm birth [20,21]. The administration of cyclooxygenase inhibitors to inhibit prostaglandin synthesis is not effective in reducing preterm birth rates [22,23]. Although oxytocin receptor antagonists and calcium channel blockers seem effective to some extent and are reported to delay preterm delivery for several days, the majority of women (~65%) receiving this treatment still deliver preterm [23,24]. No pharmacological treatment thus far has been shown to significantly prolong pregnancy or improve neonatal outcome [25]. As none of the currently available treatment options have been successful in substantially reducing preterm labour, it is necessary to identify novel molecular targets that govern the activation of the inflammatory pathways and progesterone withdrawal. This will provide new rational therapeutic targets to prevent preterm labour. The lack of access to human myometrial samples during ongoing uncomplicated pregnancy seriously hampers proper understanding of the sequence of events leading to the initiation of parturition.

To address this and understand the sequential order of molecular events, in our previous work we used mouse as a model and profiled gene expression in mouse uterus from early embryonal day (E) 6.5 to late gestation E17.5. During mid-gestation (E10.5, E12.5, E15.5) we identified Tbx2 as one of the putative upstream regulators preceding progesterone withdrawal [26]. TBX2 is a member of the T-box family of transcription factors functioning mainly as a transcriptional repressor [27,28]. The role of TBX2 has been widely studied in development of the heart and the mammary gland, and in cancers such as breast cancer, melanoma, lung cancer, liver and prostate cancer [29,30,31]. TBX2 has never been linked to human parturition.

In the current study, we hypothesize that TBX2 represses genes that play an active role in the transition from uterine quiescence to synchronized contractions essential for parturition and we investigate the potential role of TBX2 in human myometrium. TBX2 overexpression in telomerase immortalized human myometrial cells hTERT-HM (human telomerase reverse transcriptase immortalized human myometrium) cells, known to be an accurate transcriptional model for term myometrium tissue [32], identifies targets downstream of TBX2 that are relevant for human labour. The microarray profiling of human preterm quiescent and preterm labour myometrium, combined with the additional mining of 2 publically available datasets of human myometrial term labour gene expression profiles [33,34], defines upstream regulators that relate to TBX2 expression in human myometrium and are common to both preterm and term labour.

## 2. Materials and Methods

Cell culturing: The TERT immortalized human myometrial cell line (hTERT-HM) [35] was kindly provided by J. Condon. Cells were maintained (according to the instructions from Dr. Condon; personal communication) in DMEM/F-12, GlutaMAX™ Supplement (Gibco 31331) medium with 10% Fetal calf serum (FCS) (Gibco 10270), and 1% Penicillin and Streptomycin (Gibco 15140) added as additional supplements. Cells were trypsinized at 60–70% confluency using 0.25% trypsin-EDTA (Gibco 25200-056) and subcultured 1:3 twice a week. (Gibco, Thermo Fisher Scientific, Bleiswijk, The Netherlands)

Virus production: The cDNA encoding the human TBX2 coding sequence (CDS) was extracted from pcDNA3-TBX2 construct (kindly provided by Dr. Vincent Christoffels, Amsterdam, The Netherlands), subcloned into pLenti-CMV-BLAST-DEST (Addgene, Watertown, USA, #17451) for lentiviral transduction experiments. The lentiviral expression construct pEGFPCMV-BLAST-DEST was used as mock control. Lentiviral particles were produced in HEK293T cells transiently transfected using X-treme GENE HP DNA Transfection Reagent (Roche, Almere, The Netherlands) according to the manufacturer’s recommendations.

Lentiviral transduction: 200,000 hTERT-HM cells were seeded on a 9.5 cm^2^ culture surface and transduced with 50 μL of lentiviral particles 24 h after seeding at 70–80% confluency. The medium was refreshed the next day and cells pellets and medium were harvested 3 days post transduction. QPCR and western blotting confirmed TBX2 overexpression. Culture supernatants were used for Luminex assays. TRIZOL (Thermo Fisher Scientific, Bleiswijk, The Netherlands) was used to isolate RNA and protein for micro array profiling and western blotting experiments.

Myometrium sample collection: Myometrial tissue of preterm deliveries was harvested in RNAlater (Thermo Fisher Scientific, Bleiswijk, The Netherlands) at the time of caesarean section and processed according to the manufactures instructions. In total, material and clinical data from 48 women who gave written informed consent for the study approved by the institutional review board to donate a 4 mm by 6 cm stroke of myometrium tissue from the incision site to the biobank was collected. Pregnancies with any kind of infection were excluded. Some of the preterm samples were from pregnancies complicated by preeclampsia and/or HELLP syndrome.

Sample preparation for Microarray Hybridization (tissues and cells): We selected myometrial samples from 17 preterm (<37 weeks) deliveries for microarray profiling. Of these, eight patients were not in labour (PNL) and had an iatrogenic preterm delivery. Nine patients delivered after spontaneous preterm labour (PSL). In total six hTERT-HM cell cultures from three independent experiments, three overexpressing TBX2 (pTBX2-CMV-BLAST-DEST transduced) and three control (pEGFPCMV-BLAST-DEST transduced), were used for microarray profiling. Myometrial tissue samples were initially homogenized in a Magna lyser (Roche, Almere, The Netherlands) containing 1 mL of Trizol reagent. Total RNA from human myometrial tissue and cell pellets was isolated using Trizol reagent (Thermo Fisher Scientific, Bleiswijk, The Netherlands) according to the manufactures instructions. Isolated total RNA was cleaned up using RNA easy mini kit (Qiagen, Venlo, The Netherland). Labelling of total RNA with RIN > 7 on the Agilent Bioanalyser was performed using the Illumina total prep RNA amplification kit (Thermo Fisher Scientific, Bleiswijk The Netherlands) with 200 ng input. 1 μg of labelled RNA was used for array hybridization.

Microarray Hybridization: The Illumina Human HT12v4 platform was used for studying the gene expression patterns of the myometrium tissue biopsies and the transduced hTERT-HM sample set. Service XS (Leiden, The Netherlands) performed the microarray hybridization. Myometrial samples were randomized over three arrays.

Microarray data pre-processing and analysis: Data analysis was performed using Bioconductor packages (version 3.4, Bioconductor.org) using the statistical software package R (version 3.3.2, R Foundation, Vienna, Austria). The quality control was performed using array QualityMetrics Bioconductor package (built R 3.4.0, Bioconductor.org).

For the myometrial tissue samples, data analysis was performed on 17 myometrial sample arrays. On pre-processing, one preterm myometrial sample (68-PSL) was identified as an outlier based on array intensity distribution and it has been excluded from all downstream applications. Normalization was performed on 16 samples starting from the Illumina sample and control probe profiles by a normexPby-control background correction, quantile normalization, and log2 transformation (limma package version 3.32.2). Out of in total 47,231 probes, 15,431 probes with a detection *p* value of >0.05 (non-expressed) on all 16 arrays were filtered out. 

Data analysis for the transduced hTERT-HM cells was performed on six arrays from hTERT-HM transduced cells (3 TBX2 transduced vs. 3 EGFP transduced). Normalization was performed on six samples starting from the Illumina sample and control probe profiles by a normexPby-control background correction, quantile normalization, and log2 transformation (limma package). 22,023 probes with a detection *p* value of >0.05 (non-expressed) on all six arrays were filtered out.

Differential expression between the experimental conditions (Preterm labour vs. no labour, TBX2 overexpression vs. EGFP control was assessed with a moderated t test using the linear model framework (limma package). Resulting *p* values were corrected for multiple testing using the Benjamini-Hochberg false discovery rate. Corrected *p* values ≤0.05 were considered statistically significant. Probes were re-annotated using the Bioconductor IlluminaHumanv4.db package.

PubMed query for inflammatory factors involved in parturition initiation: The panel of differentially expressed inflammatory factors based on microarray analysis of TBX2 overexpressing hTERT-HM cells was extended with candidates from a PubMed query for cytokines/chemokines involved in the initiation of parturition. PubMed search string used were ((((“birth”[Title/Abstract] OR “labor”[Title/Abstract]) OR “labour”[Title/Abstract]) OR “delivery”[Title/Abstract]) AND (“preterm”[Title/Abstract] OR “pre term”[Title/Abstract])) AND “cytokines”[MeSH Major Topic] and ((((“birth”[Title/Abstract] OR “labor”[Title/Abstract]) OR “labour”[Title/Abstract]) OR “delivery”[Title/Abstract]) AND (“myometrium”[Title/Abstract] OR “uterus”[Title/Abstract])) AND “cytokines”[MeSH Major Topic]. Papers were read and scored for proinflammatory factors reported in relation to the initiation of labor. Results of the two independent searches were pooled and used to select an optimal assay panel for downstream analysis.

Luminex assay: In total 44 cytokines/chemokines were assayed using the Bio-Plex Pro 40-plex Human Chemokine Panel (171AK99MR2) (CCL1, CCL3, CCL7, CCL8, CCL11, CCL13, CCL17, CCL19, CCL20, CCL21, CCL22, CCL23, CCL24, CCL26, CCL27, CX3CL1, CXCL2, CXCL5, CXCL6, CXCL9, CXCL10, CXCL11, CXCL12, CXCL13, CXCL16, GROα, IFNγ, IL-1β, IL-2, IL-4, IL-6, IL-8, IL-10, IL-16, MCP1, MIF, MIP1δ, TECK, TNF-α, GM-CSF), HUMAN inflammation panel 1 BAFF/TNFSF13B (171BL002M), ProcartaPlex Human IL-15 Simplex IL-15 (EPX01A-12089-901), ProcartaPlex Human LIF Simplex LIF (EPX01A-10242-901), ProcartaPlex Human VCam-1 Simplex VCAM-1 (EPX01A-10232-901). All assays were performed according to the respective manufacturer’s instructions using 100 µL medium of transduced hTERT-HM cells. Fluorescence intensity was measured on a Bioplex 200 system (Bio-Rad, Veenendaal, The Netherlands) and signal quantification was performed by BioPlex manager 6.0 (Bio-Rad, Veenendaal, The Netherlands).

String Network analysis: Protein-protein interaction String network analysis was performed using dbString v10.5 (www.string-db.org, accessed on September 2018) on samples derived from transduced hTERT-HM cells with the setting of high confidence (0.700) and text mining, experiments and Databases as interaction sources. The 55 DE genes between TBX2 and control were used as input for the analysis with whole genome as the background for enrichment analysis.

Analysis of public datasets: Dataset C: Chan et al. 2014 (GSE50599) was downloaded from the Gene expression omnibus database (GEO). Data analysis was performed using Bioconductor packages (version 3.4, Bioconductor.org) using the statistical software package R (version 3.3.2, R foundation, Vienna, Austria). The RNA-Seq count dataset of five Term No Labour (TNL) samples and 5 Term spontaneous Labour (TSL) samples was imported and pre-processed using the edgeR package with the default parameters. The imported count data of 23,710 genes narrowed down to 14,588 after filtering out the zero-counts in all samples and retaining the genes with counts per million (cpm) >1 in a minimum of 4 samples. Normalization was done using the trimmed mean of M-values (TMM) provided within the edgeR package (R foundation, Vienna, Austria). The differential expression was calculated after voom transformation and following the empirical Bayes pipeline from the limma package.

Dataset D: Sharp et al. 2016 (www.ebi.ac.uk/arrayexpress/, accession number E-MTAB-3136, accessed on September 2018) was downloaded from European Bioinformatics Institute (EBI) database. This dataset was analysed as described above in the micro-array analysis section. 

Ingenuity Pathway Analysis (IPA): IPA (v 01–13, Qiagen, Venlo, The Netherlands) was used for upstream regulator analysis. As Dataset A yielded very few differentially expressed (DE) genes using an adjusted *p* value of 0.05 as cut-off. Therefore a *p* value cut off of 0.1 and log2 fold change (lfc) > 1 was used as cut-off for selection of genes for IPA analysis. In total 122 DE genes were used as input for analysis for Dataset A. For analysis of Dataset B 111 DE genes with a lfc > 1 and *p* value < 0.05 was used as input for upstream regulator analysis. From Dataset C (Chan et al. 2014) 533 DE genes with a lfc > 1 and adjusted *p* value < 0.05 were used as input for the upstream regulator analysis. DE analysis of Dataset D (Sharp et al. 2018), yielded only 24 DE genes using a lfc cut-off > 1, hence a less stringent cut-off of lfc >0.5 was used which yielded 379 genes for IPA input. Upstream regulators with an activation Z score >2 were considered significant. The top 50 upstream regulators identified in independent analyses were used to generate network plots.

Reverse transcription quantitative polymerase chain reaction (qPCR): cDNA synthesis was performed using the Roche (Almere, The Netherlands) First Strand cDNA Synthesis Kit for RT-PCR (AMV) with 200 ng as total RNA input, primed using random primers according to manufacturer’s instructions. QPCR was performed with the SYBR green assay (LightCycler^®^ 480 SYBR Green I Master, Roche, Almere, The Netherlands) using the TBX2 forward 5′-GACAAGCACGGCTTCACC and reverse primer 5′-GTTGGCTCGCACTATGTGG and 2.5 μL of myometrial cDNA as input. The experiment was run on a Roche LightCycler^®^ 480 System (Roche, Almere, The Netherlands). Expression of TBX2 was normalized using the geometric mean of reference genes PSMD4 (forward: 5′-GGCAAGATCACCTTCTGCAC, reverse: 5′-CTTCCCACAAAGGCAATGAT), GUSB [36,37] (forward: 5′-GGAGTGCAAGGAGCTGGAC, reverse: 5′-ATTGAAGCTGGAGGGAACTG) and ALU [38] (forward primer: 5′-CATGGTGAAACCCCGTCTCTA, reverse primer: 5′-GCCTCAGCCTCCCGAGTAG).

Western Blotting: Lysates from transduced hTERT-HM cells were investigated by Western Blot analyses. Protein concentration in the supernatant was determined by the Pierce BCA protein assay (Thermo Fisher Scientific, Bleiswijk, The Netherlands). Equal amounts of protein samples were separated by SDS-PAGE and transferred to an Immobilon-Fl PVDF-membrane (0.45 µm). After blotting the membranes were stained with the REVERT total Protein Stain_926-11010 (Li-Cor, Lincoln, USA) procedure according to the manufacturer’s instructions. For TBX2 protein estimation, blots were either incubated with anti-TBX2 (Santa Cruz Biotechnology SC-17880, 1:200, Heidelberg, Germany) primary antibody, followed by GAMIRDye 800 Goat-anti-mouse (Li-Cor) secondary antibody according to instructions of the manufacturer. Lysates from transduced hTERT-HM cells were additionally investigated by Western Blot analyses using antibodies specific for NFκB p65 (Santa Cruz Biotechnology antibody sc-8008, 1:200), RelB p68 (Santa Cruz Biotechnology antibody sc-48366, 1:200), Progesterone receptor A and B isoforms (PR-A 81 kDa and PR-B 116 kDa: Santa Cruz Biotechnology antibody sc-166169, 1:100), the oestrogen receptorα (Erα: Santa Cruz Biotechnology antibody sc-8002, 1:200), Oxytocin receptor (Santa Cruz Biotechnology antibody sc-33209, 1:200) and Connexin-43 (BD transduction Laboratories antibody 610062, 1:250, Vianen, The Netherlands). Protein quantification was done using the REVERT^TM^ Total Protein Stain Normalization with imaging Studio Lite version 5.2 software (Li-Cor, Lincoln, USA) and all protein data are normalized to revert staining imaged at 700 nm.

## 3. Results

### 3.1. TBX2 Downregulates TNFα and Interferon Signaling in Myometrial Cells

#### 3.1.1. Microarray Analysis of hTERT-HM Cells with TBX2 Overexpression Compared to Control

**Figure 1:**
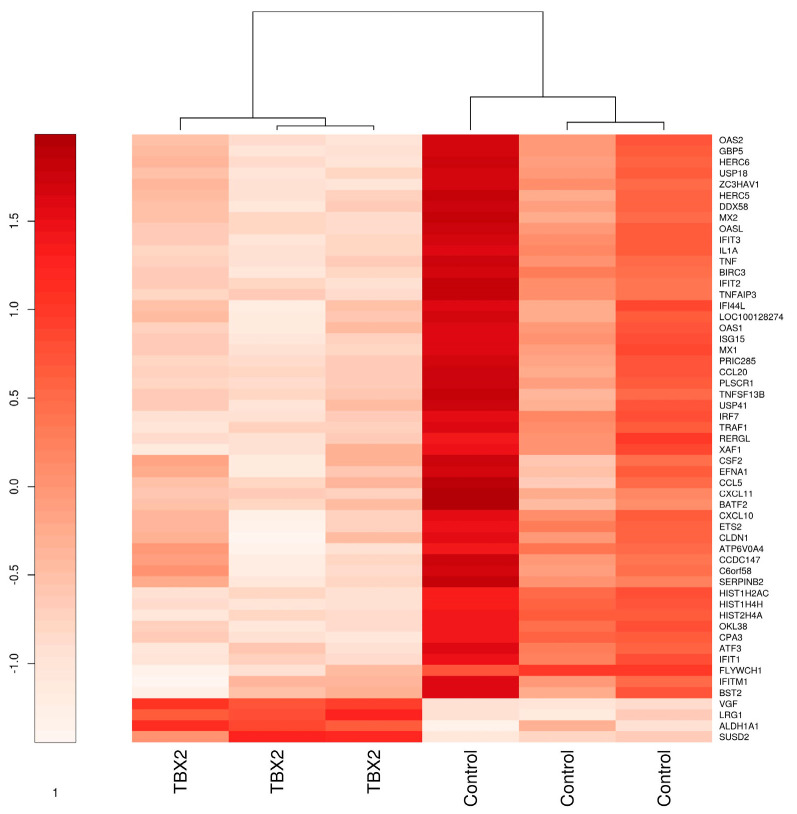
Heatmap of Differentially expressed genes in hTERT-HM cells overexpressing TBX2.

In order to comprehend the role of TBX2 in human myometrium during pregnancy and labour and determine its downstream targets in relation to their potential role in the initiation of parturition, we overexpressed TBX2 in telomerase immortalized human myometrial cells (hTERT-HM). Overexpression of TBX2 protein was confirmed by Western blot analysis. RNA microarray analysis using a Benjamini Hochberg adjusted *p* value < 0.05 identified eight differentially expressed (DE) genes (VGF, FLYWCH1, HIST1H4H, HIST1H2AC, LRG1, HIST2H4A, ALDH1A1). Applying a *p* value < 0.1 and log2 fold change (lfc) >1.5 cut-off identified a set of 55 DE genes of which 51 genes are downregulated (ATF3, ATP6V0A4, BATF2, BIRC3, BST2, C6orf58, CCDC147, CCL20, CCL5, CLDN1, CPA3, CSF2, CXCL10, CXCL11, DDX58, EFNA1, ETS2, FLYWCH1, GBP5, HERC5, HERC6, HIST1H2AC, HIST1H4H, HIST2H4A, IFI44L, IFIT1, IFIT2, IFIT3, IFITM1, IL1A, IRF7, ISG15, LOC100128274, MX1, MX2, OAS1, OAS2, OASL, OKL38, PLSCR1, PRIC285, RERGL, SERPINB2, TNF, TNFAIP3, TNFSF13B, TRAF1, USP18, USP41, XAF1, ZC3HAV1) and four genes are downregulated (ALDH1A1, LRG1, SUSD2, VGF) in case of TBX2 overexpression compared to the mock transduced cells (Table S1). Hierarchical clustering of the 55 differentially expressed genes results in two separate clusters, which are represented as a heat map in Figure 1.

Differentially expressed (*n* = 55) genes with a log2 fold change >1.5 (*p* value < 0.1) in TBX2 overexpressing hTERT-HM myometrial cells vs. EGFP transduced control cells. Genes are hierarchically clustered using the ward.D algorithm.

**Figure 2:**
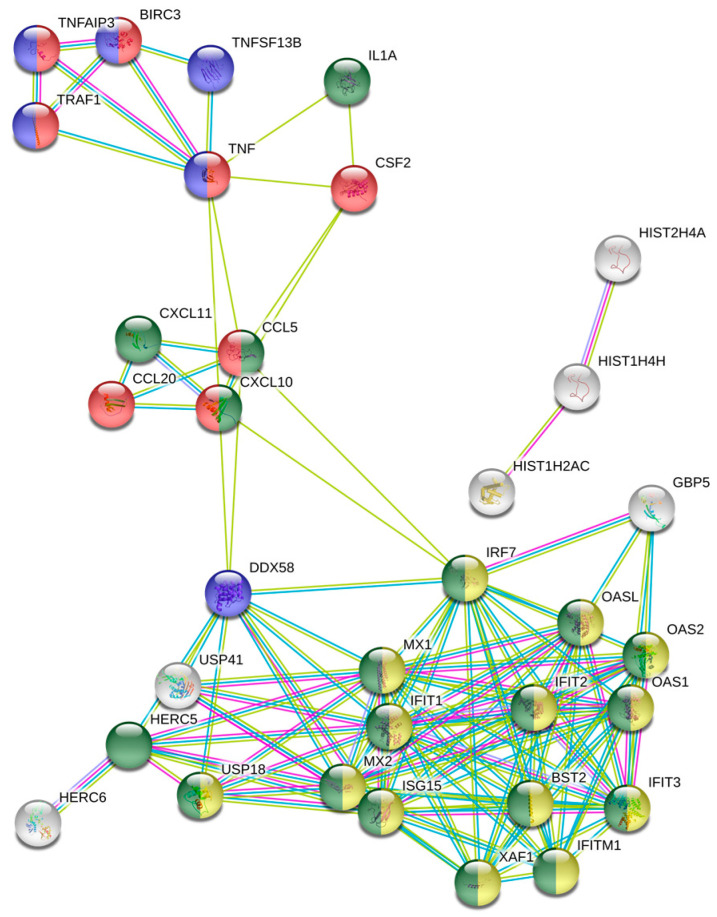
String network analysis of TBX2 induced differentially expressed genes.

To investigate the relationship between the differentially expressed genes in terms of protein-protein interactions, we performed string network analysis with a setting of high confidence (0.700) and text mining, experiments and databases as interaction sources. The resulting protein-protein interaction (PPI) network describing the relation between the differentially expressed genes is depicted in Figure 1 with 128 interactions with a *p* value < 1.0 × 10^−16^. (Figure 2). For pathway analysis, the KEGG pathway analysis module provided within the dbstring platform was utilized to check for pathways that are significantly enriched with our provided differentially expressed genes. Only the KEGG enrichments with the term “pathway” in their name description were considered in our analysis. Our results indicate that TNF signalling pathway (*p* value 1.14 × 10^−7^) and NF-kB signalling pathway (*p* value 9.36 × 10^−6^) are the top 2 significantly enriched pathways (Table 1). However, there was a distinct interlinked cluster of genes (bottom cluster of genes Figure 2) on String analysis that did not come up in the list of enriched KEGG pathways. Hence we looked at the Gene ontology (GO) enrichment module that indicates that the bottom cluster of genes (Figure 2 highlighted in yellow and green) are enriched in GO Type I Interferon signalling pathway (*p* value 2.45 × 10^−20^) and GO cytokine mediated signalling pathway (*p* value 3.98 × 10^−17^). (Table 2) Based on their lower expression in the hTERT-HM cells TBX2 overexpressing group, this indicates a downregulation of the associated pathways.

Stringv10.5 network analysis was performed on differentially expressed genes (log2 fold change >1.5; *p* value < 0.05) in hTERT-HM cells overexpressing TBX2 compared to control with the settings of high confidence (0.700) combined with Text mining, Experiments and Databases as interaction sources. Circle colours indicate enrichment in specific pathways. Red circles: KEGG TNF signalling pathway (*p* value 9.74 × 10^−8^), yellow circles: KEGG NF-kB signalling pathway (*p* value 8.37 × 10^−6^), green circles: GO Type I interferon signalling pathway (*p* value 2.45 × 10^−20^), blue circles: GO Immune response (*p* value 1.52 × 10^−17^). Line colours indicate interaction identified via Experiments (pink), Databases (blue) and Text mining (yellow).

#### 3.1.2. Cytokine/Chemokine Release after TBX2 Overexpression in hTERT-HM Cells

**Figure 3:**
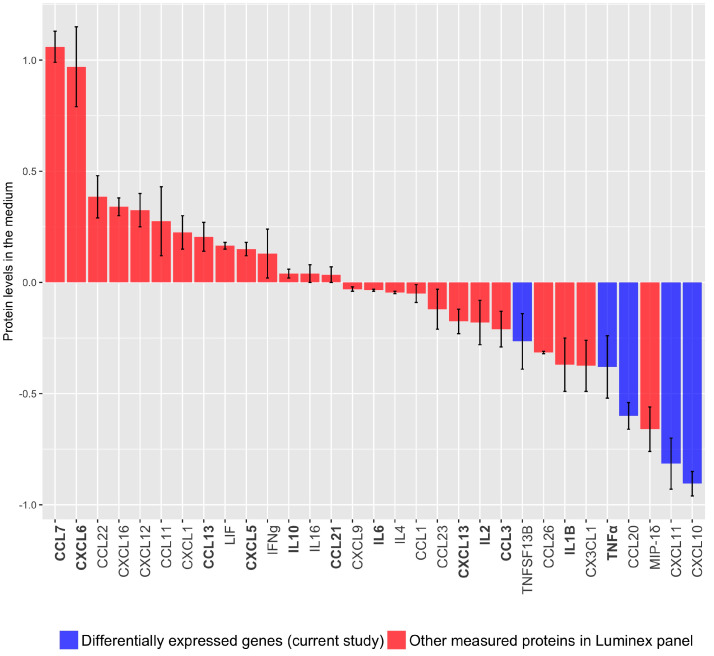
Cytokines and chemokines regulated by TBX2 overexpression. In bold: cytokines/chemokines involved in the initiation in parturition based on PubMed query.

To validate these findings on the protein level, we measured cytokine/chemokine release to the medium in hTERT-HM cells expressing TBX2. The cytokine/chemokine panels used were selected based on our own micro-array profiling of TBX2 overexpressing hTERT-HM cells and a number of proteins reported in relation to the transition from uterine quiescence to overt labour based on a PubMed query (see material and methods). Consistent with the findings on our microarray expression profiling, CXCL10, CXCL11, CCL20 and TNFα release is decreased in hTERT-HM cells overexpressing TBX2 compared vs. control (Figure 3). Additionally, CCL7, CXCL6, CCL13 known to play a role in parturition initiation, are higher in TBX2 overexpression versus control, while TNFα, IL1β, CCL3, IL2 and CXCL13 levels are lower. CXCL5, CCL21 and IL6, previously reported in relation to parturition do not seem to be under TBX2 control. Levels of CCL24, GM-CSF and IL-15 were below the assay detection level.

Represented are the cytokine and chemokine protein levels secreted from hTERT-HM cells in response to altered TBX2 expression and determined by Luminex assays. The bars are sorted from high to low levels of protein present in the culture medium. Protein levels in medium from mock-transduced control cells are set at zero. Error bars represent the standard error of the mean of two independent experimental observations.

Purple bars: Differentially secreted proteins in hTERT-HM cells overexpressing TBX2 vs. control identified by microanalysis in the current study. Red bars: Proteins co-measured because of their presence on the arrays used. Bold *x*-axis labels: previously reported in relation with parturition and identified by PubMed query (see Material and Methods).

### 3.2. TBX2 Inhibits Crucial Upstream Regulators in Preterm and Term Labour

#### 3.2.1. TBX2 mRNA Expression in Human Preterm Myometrium Samples

**Figure 4:**
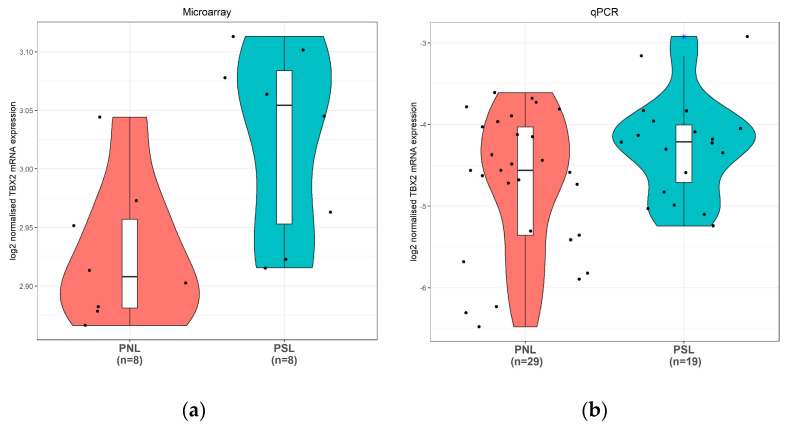
TBX2 mRNA expression in human preterm myometrial samples. (**a**): TBX2 mRNA expression measured by Illumina microarray profiling (* *t*-test *p* value < 0.05). (**b**): TBX2 mRNA expression measured by quantitative polymerase chain reaction (qPCR) (* *t*-test *p* value < 0.05). Dots indicate individual data points. The width of the density curves corresponds to the frequency of the observations. The box and whisker plot in the center with a median represents the interquartile range and the 95% confidence intervals. PNL: Preterm no labour; PSL: Preterm spontaneous labour.

Microarray profiling of preterm no labour (PNL) and preterm spontaneous labour (PSL) myometrium samples used shows a statistically significant (*p* = 0.015) upregulation of TBX2 mRNA in contractile myometrium (Figure 4a). QPCR analysis on an increased number of samples confirmed this finding (*p* = 0.035) (Figure 4b).

The clinical information of the sample subset used for microarray analysis is provided in Table 3. The characteristics spontaneous contractions, contractions at any stage during pregnancy and hypertension during pregnancy are statistically different between groups and inherent to the group definitions. In the PSL group, a caesarean section is indicated because of fetal or maternal complications while an iatrogenic preterm delivery by caesarean section is performed because of pregnancy complications such as hypertension (Detailed clinical information in Table S2).

#### 3.2.2. TBX2 mRNA Expression in Human Term Myometrium Samples

**Figure 5:**
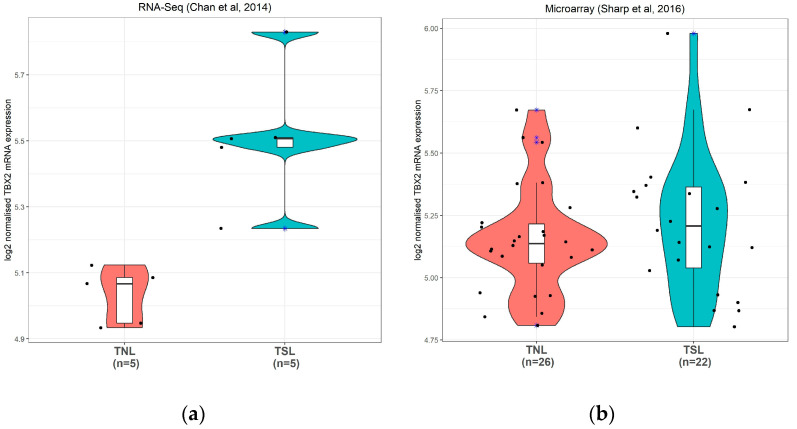
TBX2 mRNA expression in human preterm myometrial samples. (**a**): TBX2 expression in human myometrium by RNA-Sequencing (extracted from Chan et al., 2014). (**b**): TBX2 expression in human myometrium by microarray profiling (extracted from Sharp et al., 2016). A blue asterisk marks outliers. Dots indicate individual data points. The width of the density curves in the violin plot corresponds to the frequency of the observations. The box & whisker plot in the center with a median represents the interquartile range and the 95% confidence intervals. TNL: Term no labour; TSL: Term spontaneous labour. * *t*-test *p* value < 0.05.

To investigate the role of TBX2 in term labour we used two publicly available datasets, from here on referred to as Dataset C containing RNA sequence profiles (Chan et al., 2014; RNA-Seq) [33] and Dataset D containing profiles generated by microarray analysis (Sharp et al. 2016; Microarray) [34]. Dataset C showed a significant increase in TBX2 expression in myometrium in TNL (*n* = 5) compared to TSL (*n* = 5) but the difference in TBX2 expression in Dataset D is not significantly different between the TNL and TSL groups (Figure 5).

#### 3.2.3. The Putative Role of TBX2 in Myometrium during Labour

To generate a comprehensive overview of TBX2 regulation in aspects of preterm and term labour we utilized the DE genes identified in each dataset, Dataset A (TBX2 overexpression-current study), Dataset B (Preterm labour and preterm no labour myometrial microarray profiles; current study), Dataset C (Term labour/no-labour myometrial next generation sequencing profile by Chan et al.) [33] and Dataset D (Term myometrial labour/no-labour microarray profile-Sharp et al.) [34] and performed upstream regulator analysis using the Ingenuity Pathway Analysis (IPA) application.

**Figure 6:**
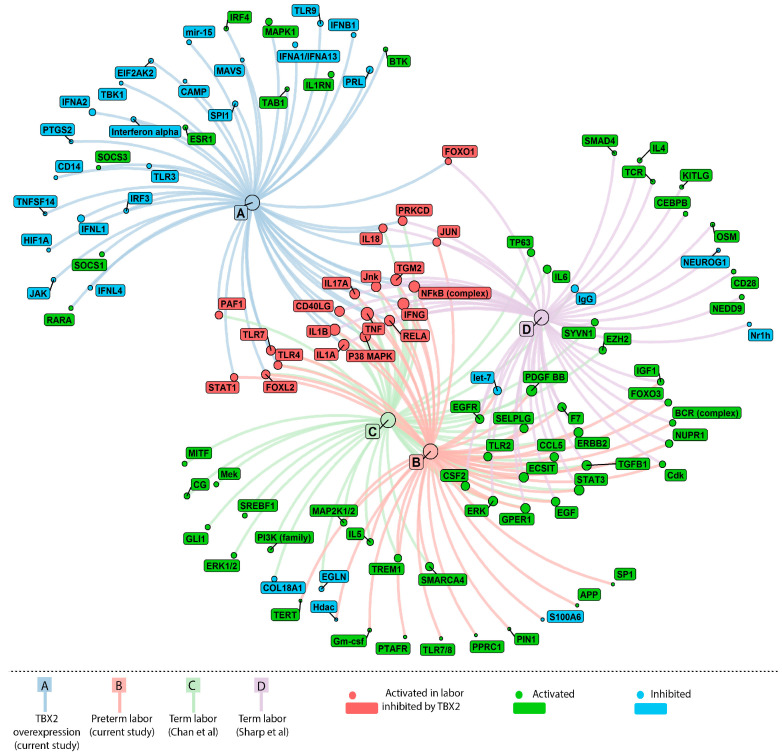
Upstream regulators governing preterm and term labour.

**Figure 7:**
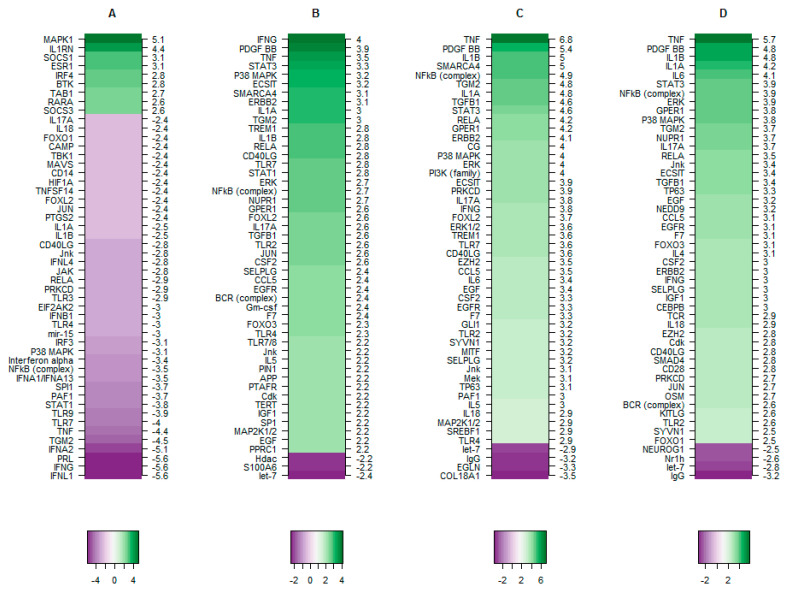
The Z-scores of the top 50 upstream regulators. The top 50 upstream regulators identified in study (**A**–**C**) are represented as a network plot. The four central nodes (**A**–**D**) and their corresponding edges (the coloured lines) represented in this figure correspond to four independent datasets. Dataset A: hTERT-HM cells overexpressing TBX2 (light blue lines, source current study).Dataset B: Preterm Labour in human myometrium (pink lines, source current study).Dataset C: Term labour in human myometrium (light green lines, source Chan et al., 2014). Dataset D: Term labour in human myometrium (purple lines, source Sharp et al., 2016).

The top 50 upstream regulators (Table S3) identified in each dataset analysed independently is represented as a network plot in Figure 6 and the Z scores of the predicted upstream regulators are illustrated as a heat map in Figure 7. TBX2 overexpressing hTERT-HM cells (Dataset A) show relatively more inhibited upstream regulators than activated, whereas in Preterm labour (dataset B) and Term labour (C and D) the vice versa holds true (Figure 7). Based on IPA analysis TNF, IFNG, IL1B, IL1A, NFkB (complex), TGM2, P38 MAPK, RELA, CD40LG, IL17A, JNK are present as activated regulators in all 3 preterm and term labour datasets (Datasets B, C, D) but are inhibited by TBX2 overexpression (Dataset A) (Figure 6). Of the identified upstream regulators, TNF is identified as the top regulator with the highest activation score in both the term labour datasets (Dataset C and Dataset D) and third highest ranked upstream regulator in preterm labour dataset, after IFNG and PDGFBB (Figure 7). The well-established inflammatory regulator of labour RELA is present as an active regulator in all 3 labour datasets (preterm and term B, C, D) but is identified as an inhibited regulator in TBX2 overexpression (Figure 6 and Figure 7).

Circles represent upstream regulators/nodes and squares contain their respective labels. Green circles/squares represent activated upstream regulators and blue circles/squares represent the inhibited upstream regulators in myometrium in labour.

Red coloured nodes/squares represent regulators activated in labour and inhibited by TBX2 overexpression in myometrium cells. Node size positively correlates with absolute Z score values (higher Z-score is indicated by bigger node size).

The Z-scores of top the 50 upstream regulators identified in each study is represented as a heat plot sorted in descending order. A: regulators identified in TBX2 overexpression hTERT-HM cells (dataset current study), B: regulators identified in preterm labour human myometrium samples (data set current study), C: regulators identified in term labour human myometrium samples (dataset et al., 2014), D: regulators identified in term labour human myometrium (Dataset Sharp et al., 2016). Green: activated and Purple: inhibited upstream regulators.

The Preterm labour dataset B has STAT1 as a unique activated upstream regulator that is absent in term labour (datasets C D) and is inhibited by TBX2 overexpression (dataset A). The other unique activated upstream regulators in preterm labour (dataset B) include SP1, APP, PIN1, PPRC1, TLR7/8, PTAFR, Gm-csf, and TERT. The activated regulators specific for term labour datasets B and C include TP63, IL6, SYNV1, and EZH2.

The top inhibited upstream regulators that are present only in TBX2 overexpression dataset (Dataset A) include IFNL1, PRL, IFNA2, TLR9 and SPI1. It is interesting to note that PTGS2, a regulator commonly implicated in parturition initiation and maintenance of pregnancy [39] is present as an inhibited regulator of the genes associated with TBX2 overexpression.

Western blot analysis to determine additional downstream effects of TBX2 overexpression in hTERT-HM cells compared to control on the protein level, shows that TBX2 overexpression significantly correlates with decreased levels of the p68 RelB protein, the oxytocin receptor and connexin43 while the oestrogen receptor alpha seems upregulated although not statistically significant. (Table 4, Figure S1).

## 4. Discussion

Our previous study on mouse uterine gene expression during gestation identified Tbx2 as one of the top upstream regulators in pregnant mice uterus during mid-gestation preceding progesterone withdrawal [26]. TBX2 is strongly associated with development of heart and limbs and cancer [40,41,42]. TBX2 expression in human and mouse mammary gland and mouse gonads and the genital ducts has been established and its expression in uterus has been listed [30,43,44]. Based on its function as transcriptional repressor [45] we hypothesized that TBX2 represses genes that play an active role in the transition from uterine quiescence to synchronized contractions essential for parturition. In the current study, we investigated the function of TBX2 in a human myometrial immortalized cell line hTERT-HM, where we upregulate TBX2 expression using lentiviral transduction and investigate the expression of TBX2 in human myometrium at the time of delivery.

RNA-microarray analysis of hTERT-HM cells overexpressing TBX2 demonstrates downregulation of genes mainly involved in the TNF signalling and Type I interferon-signalling pathways. On the protein level, we confirm this by showing downregulation of CXCL10, CXCL11, CCL20, TNFSF13B and TNFα protein levels in TBX2 overexpressing hTERT-HM cells compared to the mock-transduced cells. It has been shown that immune cells invading into the myometrium secrete proinflammatory cytokines and chemokines that activate proinflammatory transcription factors such as NFκb [46,47]. Here we show that myometrium cells themselves are also able to secrete these proinflammatory cytokines and chemokines. In a recently published systematic review, TNFα has been reported as a biomarker expressed in multiple gestational tissues including the myometrium in term labour [18].

Apart from reduced levels of CXCL11, CCL20, TNFSF13B and TNFα in hTERT-HM cells overexpressing TBX2, also reduced levels of IL1B, CCL3, IL2 and CXCL13 and increased levels of CCL7, CXCL6, CCL123 and CXCL5 clearly are downstream effects of TBX2 expression. 

Further bioinformatical analysis of our own preterm labour dataset (Dataset B) and the 2 public datasets of term labour (Dataset C and Dataset D) shows that the differentially expressed genes in all 3 datasets share a number of common upstream regulators (Table S3 and Figure 6). Interestingly, a number of the upstream regulators common to both preterm and term labour are under control of TBX2 expression (Dataset A) and are depicted in the red text boxes in Figure 6. The majority of them have already been reported in relation to labour. Myometrial activation of AP1 (consisting of c-Jun and c-Fos) via Jnk is known to drive the production of cytokines, Cox-2, and connexin43, and drives the inflammatory pathways that cause preterm labour [48]. Specific inhibition of JNK has been reported to delay preterm labour [49]. Telomere dependent and p38 mitogen activated protein kinase (MAPK) induced activation of senescence plays a role in physiologic aging of fetal tissues, a potential mechanistic feature of normal parturition. This premature aging is associated with sterile inflammation capable of triggering preterm labour or preterm premature rupture of membranes [50]. Platelet-activating factor (PAF) is a potent pro-inflammatory phospholipid secreted into amniotic fluid with fetal long surfactant near term. It has been suggested that PAF of fetal lung origin may promote myometrial contractility [51,52]. Parturition in rat can be prolonged with treatment of a PAF receptor antagonist showing that PAF is an integral mediator in initiating myometrial contractions [53]. STAT1, signal transducer and activator of transcription 1, has been associated with preterm premature rupture of membranes [54]. TLR-4 and -7 are expressed in maternal neutrophils and polymorphisms are associated with spontaneous preterm labour [55,56]. FOXO1 is a pro-inflammatory modulator linked to expression of IL-1B, IL-6, and IL-8 [57]. RELA and CD40LG are members of the NF-kB family that regulate the expression of a range of pro-inflammatory mediators (TNF, INFG, IL-1A, IL-1B, IL-4, IL-6, and IL-18) whose involvement in the transition from uterine quiescence to synchronized contractile state has been well established [58,59]. Based on the IPA prediction in the current study they are under control of TBX2 and their downstream effects as expression of RelB, the oestrogen receptor alpha and connexin43 have additionally been confirmed at the protein level.

FOXL2, PRKCD and TGM2 have not been reported in relation to labour previously. In the current study we identify FOXL2, PRKCD and TGM2 as factors activated in both preterm and term labour that are under control of TBX2.

Combining the data from TBX2 overexpression in myometrium cells with previously published datasets on term myometrium and hTERT-HM cells overexpressing TBX2 show that a number of factors that are activated in labour are inhibited by TBX2. TBX2 functions upstream of previously identified pathways essential for the transition of uterine quiescence to synchronized contraction necessary for delivery in human myometrium.

Based on this, TBX2 activity in both preterm and term quiescent myometrium should be increased compared to that in labour. This is not what we see on the mRNA level in preterm samples and also not in the datasets of term samples. (Figure 4 and Figure 5) Currently we have no explanation for this other than that probably the mRNA levels and transcriptional activity of TBX2 in human myometrium are not correlated. The discrepancy could also be explained by epigenetic regulation influencing the protein levels translated from a set of TBX2 mRNA molecules, as the TBX2 promoter region is relatively rich in CpG islands [60]. Activity of the TBX2 transcription factor will be subject to the presence of specific currently not-identified co-factors that might be a rate-limiting factor. For *Caenorhabditis elegans*, it has been reported that TBX2 can directly regulate its own expression in a negative autoregulatory loop [61]. Unfortunately, there is currently no further information on the regulation of TBX2 protein levels and activity in human myometrium.

In vitro analysis of a myometrial cell line overexpressing TBX2 and in silico analysis of the myometrial transcriptome during delivery shows that TBX2 represses a number of factors that are activated in both preterm and term labour. Future research is needed to establish the regulation processes relevant for transcriptional regulation of the TBX2 gene and functional TBX2 activity in human myometrium. More basal data on the (epigenetic) regulation of TBX2 expression and definition of TBX2 co- factors in myometrium are important. This insight will further identify putative therapeutical targets that enhance TBX2 activity and delay the transition from uterine quiescence to overt labour.

## 5. Conclusions

Microarray profiling of hTERT-HM cells overexpressing TBX2 results in downregulation of TNFα signalling and interferon signalling. This is consistent with the levels of cytokines and chemokines measured by Luminex assays in medium of the cells. In contrast, CXCL5, CCL21 and IL-6 previously reported in relation to parturition do not seem to be under TBX2 control.

Combining microarray results of our myometrium samples taken during delivery by caesarean section, the TBX2 overexpressing hTERT-HM cells compared to control, and two publicly available gene datasets comparing human term myometrium having no labour with those having spontaneous labour, identifies a subset of upstream regulators common to both preterm and term labour, activated in labour and repressed by TBX2.

We could not reproduce this in human tissue samples. In spontaneous preterm labour myometrium samples compared to samples obtained from women who were not in labour, TBX2 mRNA expression is increased. We can only explain this by speculating that the in vivo activity of TBX2 in human myometrium depends not only on the TBX2 expression levels that might be subject to epigenetic regulation but also on the levels of the accessory proteins necessary for TBX2 activity.

The potential of TBX2 as therapeutic target to treat or prevent spontaneous preterm labour as presented in the current paper merits further investigation.

## Figures and Tables

**Table 1:** KEGG pathway analysis of the differentially expressed genes.

KEGG ID	Pathway Description	Gene Count	FDR *	Matching Proteins in Network
4668	TNF signaling pathway	8	9.74 × 10^−8^	BIRC3, CCL20, CCL5, CSF2, CXCL10, TNF, TNFAIP3, TRAF1
4064	NF-kappa B signaling pathway	6	8.37 × 10^−6^	BIRC3, DDX58, TNF, TNFAIP3, TNFSF13B, TRAF1
4622	RIG-I-like receptor signaling pathway	5	3.60 × 10^−5^	CXCL10, DDX58, IRF7, ISG15, TNF
4620	Toll-like receptor signaling pathway	5	0.000199	CCL5, CXCL10, CXCL11, IRF7, TNF
4621	NOD-like receptor signaling pathway	4	0.000375	BIRC3, CCL5, TNF, TNFAIP3
4623	Cytosolic DNA-sensing pathway	4	0.000484	CCL5, CXCL10, DDX58, IRF7
4062	Chemokine signaling pathway	4	0.0276	CCL20, CCL5, CXCL10, CXCL11

* false discovery rate.

**Table 2:** GO Biological process enrichment of the Differentially Expressed genes.

GO ID Pathway	Pathway Description	Gene Count	FDR	Matching Proteins in Network
GO.0060337	type I interferon signaling pathway	14	2.45 × 10^−20^	BST2, IFIT1, IFIT2, IFIT3, IFITM1, IRF7, ISG15, MX1, MX2, OAS1, OAS2, OASL, USP18, XAF1
GO.0019221	cytokine-mediated signaling pathway	19	3.98 × 10^−17^	BST2, CCL5, CXCL10, CXCL11, HERC5, IFIT1, IFIT2, IFIT3, IFITM1, IL1A, IRF7, ISG15, MX1, MX2, OAS1, OAS2, OASL, USP18, XAF1
GO.0007166	cell surface receptor signaling pathway	22	2.18 × 10^−7^	ATP6V0A4, BIRC3, BST2, CCL5, CXCL10, CXCL11, EFNA1, HERC5, IFIT1, IFIT2, IFIT3, IFITM1, IL1A, IRF7, ISG15, MX1, MX2, OAS1, OAS2, OASL, USP18, XAF1
GO.0060333	interferon-gamma-mediated signaling pathway	4	4.74 × 10^−3^	IRF7, OAS1, OAS2, OASL
GO.2001236	regulation of extrinsic apoptotic signaling pathway	5	8.79 × 10^−3^	ATF3, CSF2, IL1A, TNFAIP3, TRAF1
GO.0002753	cytoplasmic pattern recognition receptor signaling pathway	3	1.37 × 10^−2^	DDX58, IRF7, TNFAIP3
GO.0039528	cytoplasmic pattern recognition receptor signaling pathway in response to virus	2	1.46 × 10^−2^	DDX58, IRF7
GO.0070424	regulation of nucleotide-binding oligomerization domain containing signaling pathway	2	1.91 × 10^−2^	BIRC3, TNFAIP3
GO.0034121	regulation of toll-like receptor signaling pathway	3	1.97 × 10^−2^	BIRC3, IRF7, TNFAIP3
GO.0070098	chemokine-mediated signaling pathway	3	2.16 × 10^−2^	CCL5, CXCL10, CXCL11
GO.0039535	regulation of RIG-I signaling pathway	2	3.99 × 10^−2^	BIRC3, ZC3HAV1
GO.0002221	pattern recognition receptor signaling pathway	4	4.51 × 10^−2^	BIRC3, DDX58, IRF7, TNFAIP3

**Table 3:** Clinical characteristics of patient samples used for microarray analysis.

	Preterm Spontaneous Labour (PSL) * n = 8	Preterm No Labour (PNL) * n = 8	*p* Value
Gestational age at delivery in days (mean and range)	198 (179–233)	213 (189–236)	0.10 ^#^
Gravidity (mean)	2.25	2.75	0.62 ^#^
Parity (mean)	1	0.75	0.95 ^#^
Male neonatal gender	8	5	0.2 ^##^
Neonatal weight in grams (mean and range)	1258 (820–2054)	1137 (580–1970)	0.44 ^#^
Spontaneous Contractions	6	0	<0.01 ^##^
Spontaneous Rupture of Membranes	2	0	0.47 ^##^
Contractions at any stage during pregnancy	8	0	<0.01 ^##^
Hypertension during pregnancy	0	6	<0.01 ^##^
Tocolytics during pregnancy	6	2 **	0.13 ^##^

* Unless stated otherwise; numbers reflect number of patients. ** received Nifedipine as antihypertensive drug. ^#^ Mann-Whitney U test, ^##^ Fisher’s exact test.

**Table 4:** The effect of TBX2 overexpression on labour associated proteins.

Protein	Level in TBX2 Transduced hTERT-HM Cells Compared to Mock Transduced Cells *	*p* Value **
NFκB	1.06 ± 0.20	0.694
RelB p68	0.46 ± 0.17	0.047
Progesterone receptor A isoform	-	
Progesterone receptor B isoform	0.69 ± 0.39	0.380
Estrogen receptor α	3.68 ± 0.89	0.051
Oxytocin receptor	0.40 ± 0.13	0.022
Connexin43	0.67 ± 0.04	0.006

* protein level in mock-transduced hTERT-HM cells was arbitrarily set at 1. ** Two sample unpaired *t*-test.

## Data Availability

The microarray data have been deposited in NCBI Gene Expression Omnibus and are accessible under GEO series accession number (GSE134448).

## Supplementary Materials

The following are available online at https://www.mdpi.com/article/10.3390/medicina57060515/s1, Table S1: differentially expressed genes in hTERT-HM cells with increased TBX2 expression, Table S2: clinical data, Table S3: upstream regulators in datasets A, B, C and D, Figure S1: Western blot analysis.
