# Supplementary material for: TBX2, a Novel Regulator of Labour
Source: Medicina (Kaunas). 2021 May 21;57(6):515. doi: 10.3390/medicina57060515 (PMC8224059; doi:10.3390/medicina57060515)
Supplement: Supplementary file 1 [file medicina-57-00515-s001.zip › medicina-1183368-supplementary.pdf]

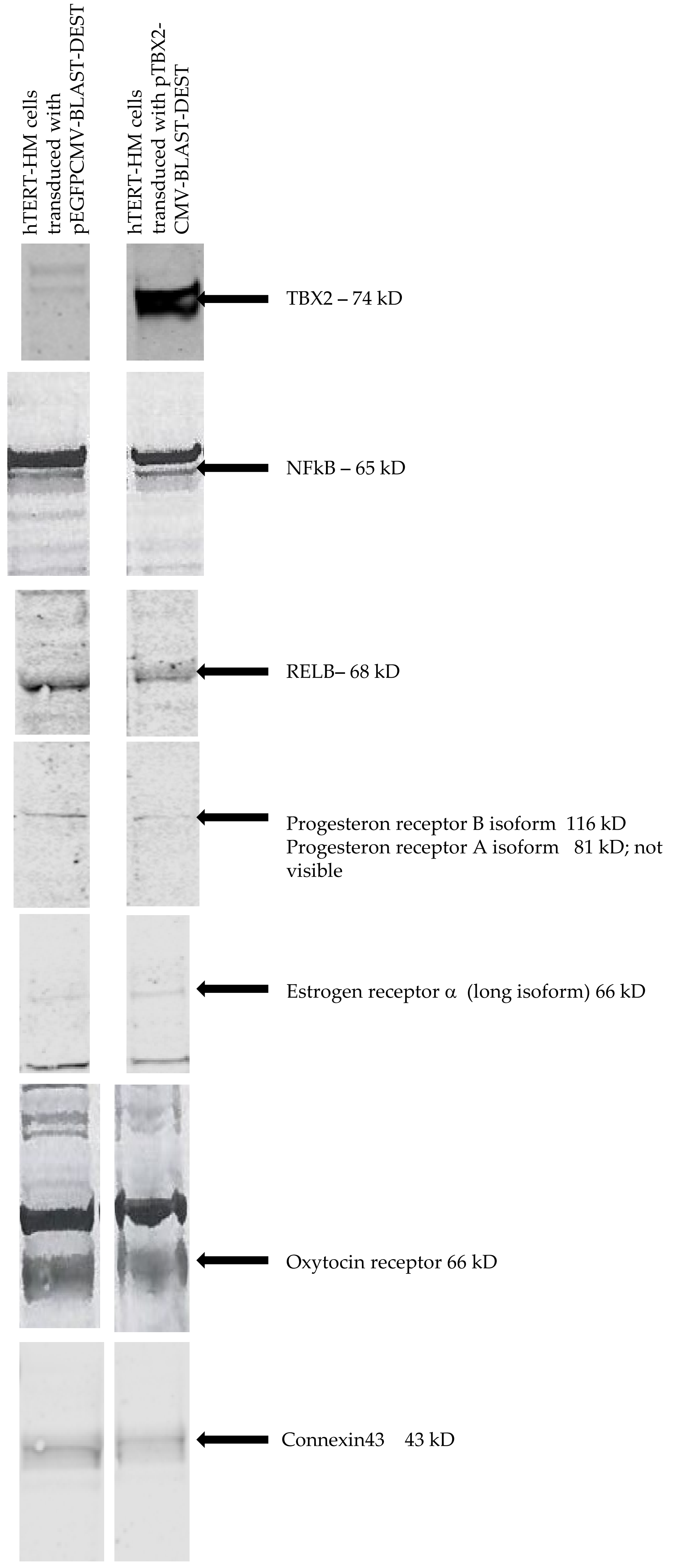

Figure S1 The effect of TBX2 overexpression on labour associated proteins
Shown are typical Western blot fragments.
For antibodies and dilutions used, see Material and Methods.
Band intensities were scanned. Comparison beween signals from mock transduced and TBX2 transduced cells was done after Revert normalisation (see Materials and Methods). The effect of TBX2 overexpression on labour associated proteins is shown in Table 4 of the manuscript.
